# Usage of UV-curable oligomer-based adhesive agent in hermatypic coral experimental research

**DOI:** 10.1016/j.mex.2019.06.007

**Published:** 2019-06-13

**Authors:** Ichiro Takeuchi, Hideyuki Yamashiro, Mikako Gushi

**Affiliations:** a Graduate School of Agriculture, Ehime University, 3-5-7 Tarumi, Matsuyama, Ehime 790-8566, Japan; b Center of Advanced Technology for the Environment, Graduate School of Agriculture, Ehime University, 3-5-7 Tarumi, Matsuyama, Ehime 790-8566, Japan; c Sesoko Station, Tropical Biosphere Research Center, University of the Ryukyus, 3422 Sesoko, Motobu, Okinawa 905-0227, Japan

**Keywords:** A new inexpensive, easy, and fast method for attaching hermatypic coral to polycarbonate bolts using an ultraviolet (UV)-curable oligomer-based adhesive agent, Attachment, UV-curable oligomer-based adhesive agent, Hermatypic coral, Inexpensive, easy, and fast method

## Abstract

The global decline of the tropical and subtropical coral reefs requires urgent completion of various experiments that will reveal the factors influencing coral health. We describe the procedure of a new inexpensive, easy, and fast method for attaching fragments of the hermatypic coral *Acropora* spp. to small polycarbonate hexagon head bolts using Bondic®, an ultraviolet (UV)-curable oligomer-based adhesive agent made by Laser Bonding Tech, Inc. (Aurora, ON, Canada). The attachment was hardened within 10 s after applying the adhesive to the cut surface of the coral fragment. The corals attached to polycarbonate bolt were tolerant to long-distance aerial transport 3 days after the attachment. In addition to its implementation in various experiments using hermatypic corals, this method will contribute to aquaculture of hermatypic corals, exhibition of corals in aquariums, and coral reef restoration.

The advantages of this new method are summarized below:

• A new UV-curable oligomer-based adhesive agent is used as an artificial substrate for coral.
• This method is inexpensive, easy to use, and coral attaches quickly to the artificial substrate.
• Corals attached to the artificial substrate can withstand long periods of transportation.

## Graphical abstract

**Figure:**
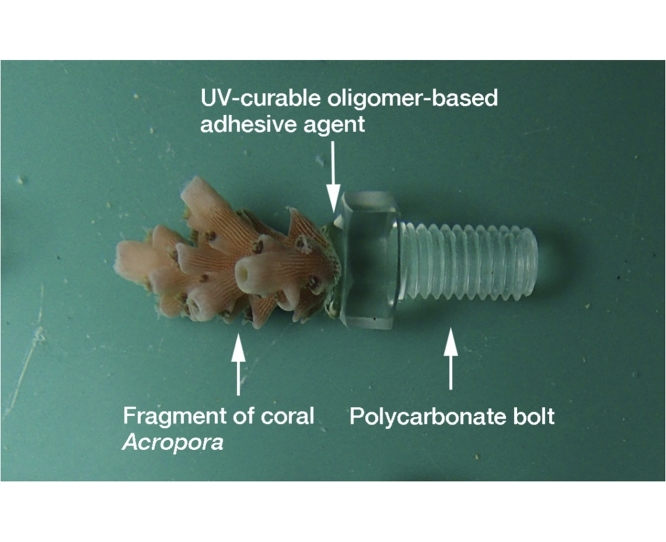

**Specifications Table**

**Table:** 

Subject Area:	Environmental Science
More specific subject area:	Ecotoxicology of hermatypic corals
Method name:	A new inexpensive, easy, and fast method for attaching hermatypic coral to polycarbonate bolts using an ultraviolet (UV)-curable oligomer-based adhesive agent
Name and reference of original method:	S. Shafir, J. Van Rijn, B. Rinkevich, Coral nubbins as source material for coral biological research: A prospectus. Aquaculture 259 (2006) 444–448. doi.org/10.1016/j.aquaculture.2006.05.026
Resource availability:	The UV-curable oligomer-based adhesive agent (Bondic®; https://notaglue.com) is commercially available. Other commercially available materials are indicated in the article.

## Method details

The global decline of the tropical and subtropical coral reefs [[1], [2], [3]] requires urgent completion of various experiments that will reveal the factors influencing coral health. While several breakthroughs in keeping hermatypic corals alive and healthy in closed aquariums were made by the mid-1980s, the difficulty in handling corals in captivity has limited their application in controlled laboratory experiments [4]. Bartlett [4] described a small-scale experimental system for various coral studies in which seawater in the experimental tanks is supplied from a seawater reservoir. He noted the usage of clippers, bonecutters, or pruning shears of various sizes for fragmenting a branching hermatypic coral such as *Acropora cervicornis* or *Pocillopora damicornis* [4]. Recently, Hirayama et al. [5] developed a hermatypic coral rearing system that uses a small aquarium without seawater supply from the reservoir.

In the 1980s and 1990s, asexual propagation of hermatypic corals became a commonplace technique in the private aquarium industry sector [6]. Various cyanoacrylate-based and epoxy glues were commonly used for propagation of hermatypic corals and fixing of cut coral fragments to substrate [6]. Borneman & Lowrie [6] noted that Surgical Simplex (Limerick, Ireland), a non-toxic calcium based surgical adhesive composed of powder and solvent, showed the best performance. This adhesive would cure completely within 5 min, and its hardness approximates that of coral skeleton.

In ecotoxicological experiments using hermatypic corals, small fragments were cut from larger parent colonies and attached to artificial substrates to be exposed to various conditions [see 4,[7], [8], [9], [10], [11], [12], [13]]. A two part epoxy polymer (Selleys Pty Ltd., Padstow, NSW, Australia) was used for sealing the base of a cut branch of *Acropora* to a substrate made of modeling clay [7,8,10]. Vijayavel & Richmond [12] used underwater cement to attach fragments of *Montipora capitata* to detachable Teflon™ plugs affixed to a tray. The cyanoacrylate-based glues, such as Super Glue 3 (Loctite, Dublin, Ireland) [4,9,11] and Aron Alpha (Toagosei Co. Ltd., Minato-ku, Tokyo, Japan) [14], have been also used in several experiments, including those of Shafir et al. [14] who described the detailed protocols for developing coral nubbins for experimental use. Similar techniques for coral fragment attachment to artificial substrate has been also used in aquaculture of hermatypic corals [see 15,16], coral reef restoration [see 17,18], and maintenance of corals in public aquaria [19].

In the present paper, we describe a new method for attaching hermatypic corals to polycarbonate hexagon head bolts using Bondic® (Laser Bonding Tech, Inc., Aurora, ON, Canada), an ultraviolet (UV)-curable oligomer-based adhesive, for coral experiments. In the past two years, Bondic® has been used for neurophysiological experiments on invertebrates to attach insects, desert ants and fruit flies, to a steel pen, tungsten wire, or filament [see [20], [21], [22], [23]].

The donor corals *Acropora digitifera* and *A. tenuis* were collected from the coast of Sesoko Island, in the northwestern part of Okinawa Island, Japan, in May to July 2018 and kept in a large aquarium (77 × 168 × 36 cm) supplied with running seawater at Sesoko Station, University of the Ryukyus, located on the south-east coast of Sesoko Island. The aquarium was set outside the research buildings under a sun-screen mesh to replicate the moderate light intensity present in the shallow coral reef environment at 3 m depth [see 24]. The sampling of *Acropora* spp. was conducted under special permission from the Okinawa Prefectural Government.

**Fig. 1:**
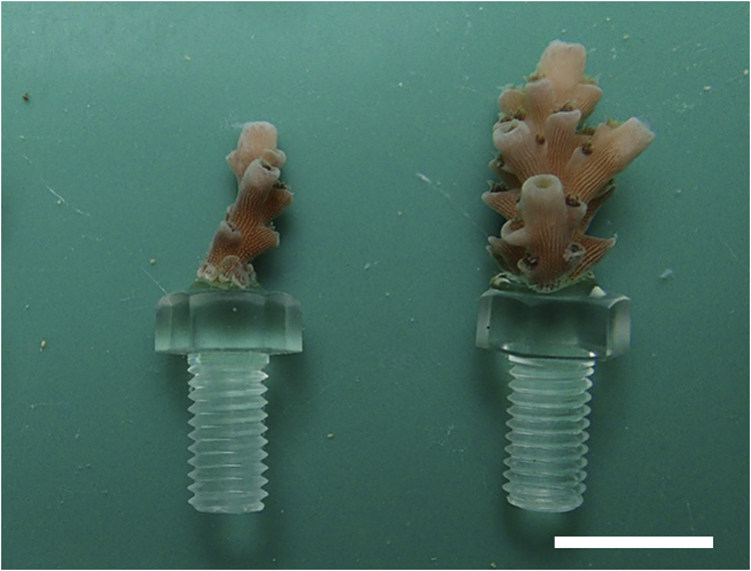
Polycarbonate hexagon head bolts with attached fragments of *Acropora digitifera*. Bar is 1.0 cm.

Three separate experimental trials were conducted from June to August 2018. Coral fragments of *Acropora digitifera* and *A. tenuis* were attached to polycarbonate hexagon head bolts using Bondic® (Fig. 1). Bondic® is an inexpensive liquid plastic, invented by a dentist turned visionary based on a dental composite [25], whose molecules connect and harden after exposure to UV light. The starters kit of Bondic®, which includes UV LED light specific for Bondic®, is <$25.00 US dollars. The material safety data sheet for Bondic® [26] lists the following chemical components: oligomer blend (50–60%), 2-hydroxy-2-methylpropiophenone, a photoinitiator of the UV radiation curable process (1–10%), photoinitiator (no description of its chemical identity) (1–10%), and monomer blend (30–40%). Table 1 lists the material required for cutting the donor hermatypic colonies of *Acropora digitifera* and *A. tenuis* and attaching the cut fragments to polycarbonate hexagon head bolts using Bondic®.

**Table 1:** List of material used for cutting the donor hermatypic colony of *Acropora* spp. and attaching coral fragments to polycarbonate bolts using Bondic®.

1	Plastic seawater tank (65 × 110 × 18 cm)
2	Diagonal cutting pliers, 110 mm in total length (MP4-110; Fujiya Co., Ltd., Higashiosaka, Osaka, Japan) and 150 mm in total length (FPN-150FS; Fujiya Co., Ltd., Higashiosaka, Osaka, Japan)
3	18–8 stainless steel forceps, 250 mm in total length (K-18-250; Kowa Forceps Industry Co., Ltd., Katsushika-ku, Tokyo, Japan)
4	Ultraviolet (UV)-curable oligomer-based adhesive with specific UV light (Bondic®; Laser Bonding Tech, Inc., Aurora, ON, Canada)
5	Sterilized filter paper (Kimwipes®; Nippon Paper Crecia Co., Ltd., Chiyoda-ku, Tokyo, Japan)
6	Polycarbonate hexagon head bolts (PCBT-0510; Wilco Inc., Yokohama, Kanagawa, Japan)
7	UV cut glass (YX-540; Yamamoto Kogaku Co., Ltd, Higashiosaka, Osaka, Japan)
8	Disposable gloves (8-4053-01/8-4053-02/8-4053-03; As One Corp., Osaka, Osaka, Japan)
9	Mesh substrate for polycarbonate screws; made of thermoplastic fluoropolymer screen (mesh size, 2.14 mm; F-3056-008; Flon Industry, Bunkyo-ku, Tokyo Japan), ethylene-vinyl acetate (EVA) screen (mesh size, 7.5 mm; N-523; DAIPLA Corp., Osaka, Osaka, Japan), plastic square tube, and 18-8 stainless steel forceps (used for weight)

The procedure is summarized below.

**Fig. 2:**
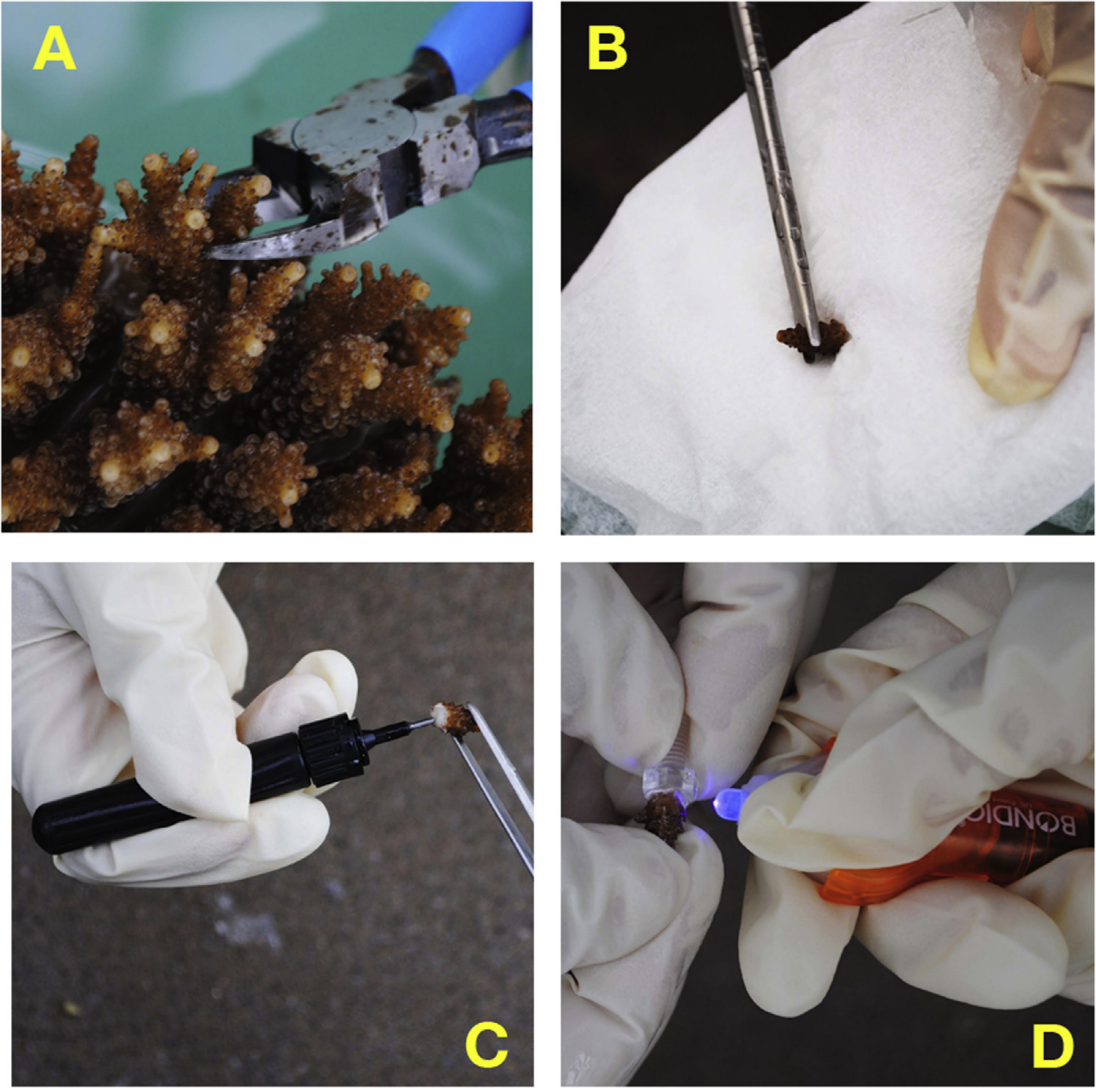
Illustration of the protocol followed to attach *Acropora* spp. coral fragments to polycarbonate hexagon head bolts using Bondic®. A, cutting a branch of donor coral, *Acropora digitifera*. B, removing moisture from the cut surface of the coral branch. C, applying Bondic® to the cut surface of the coral branch. D, irradiating the area with applied Bondic® with ultraviolet LED radiation.
3) The cut fragments were kept in an aquarium (65 × 110 × 18 cm) until use.
4) The cut coral fragment was removed from the aquarium with forceps, and moisture was absorbed from the cut section with sterilized filter paper (Fig. 2B).
5) A small drop of Bondic® was applied to the cut section of the coral fragment (Fig. 2C), and the fragment was immediately attached to the top of a polycarbonate hexagon head bolt.
  6) To prevent hardening of the polymer due to sunlight radiation, this procedure should be conducted in a shaded area or in a room.
7) The UV radiation LED provided with Bondic® was irradiated around the attachment for ca. 5–10 s (Fig. 2D).
8) The polycarbonate screw with attached coral fragment (Fig. 1) was kept in a shallow aquarium.
9) The coral fragments mounted on polycarbonate hexagon head bolts were screwed into a mesh substrate. The mesh substrate consisted of two screen layers, a thermoplastic fluoropolymer screen (top layer) with the 2.14 mm mesh size and an ethylene-vinyl acetate (EVA) screen (base) with the 7.5 mm mesh size (Fig. 3). The two screens were fixed together on plastic square tubes by small cable ties, and 18-8 stainless steel forceps were set inside the square tubes for weight to prevent the screens from floating.

1) The donor coral was moved from a large aquarium (77 × 168 × 36 cm) supplied with running seawater to a relatively shallow aquarium also outfitted with running seawater.
2) Using either size of a diagonal cutting plier, the tip of each branch-like part (ca. 1.0–1.5 cm in length) of a donor coral was cut as cleanly as possible to minimize damage to the coral (Fig. 2A).

**Fig. 3:**
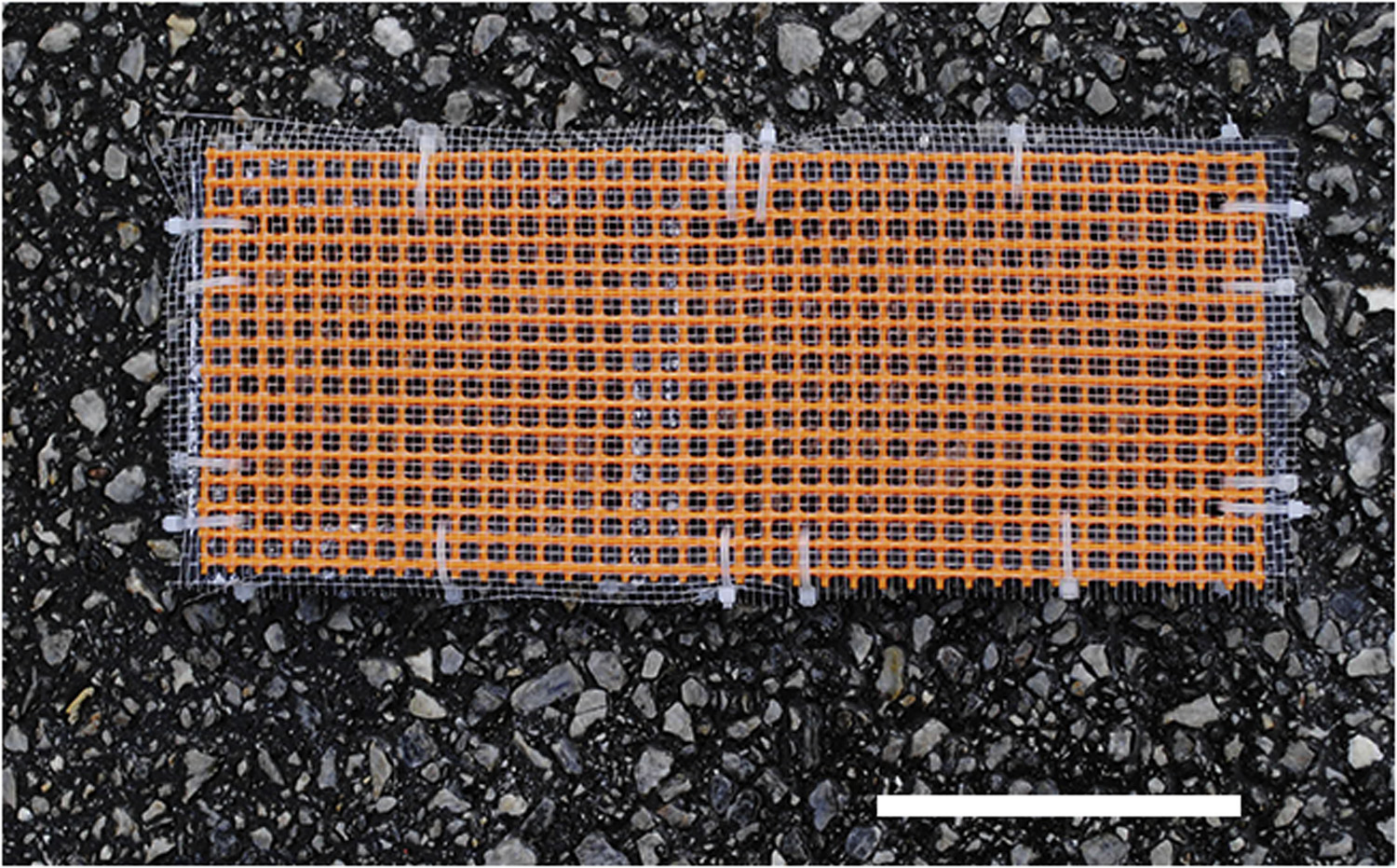
The mesh substrate consisted of two screen layers, a thermoplastic fluoropolymer screen (top layer) and an ethylene-vinyl acetate (EVA) screen (base). Bar is 10 cm.
10) The openings of the top mesh layer were widened to facilitate screwing of the bolts into the mesh substrate.
11) The substrate was transferred to a larger aquarium supplied with running seawater (Fig. 4A).

**Fig. 4:**
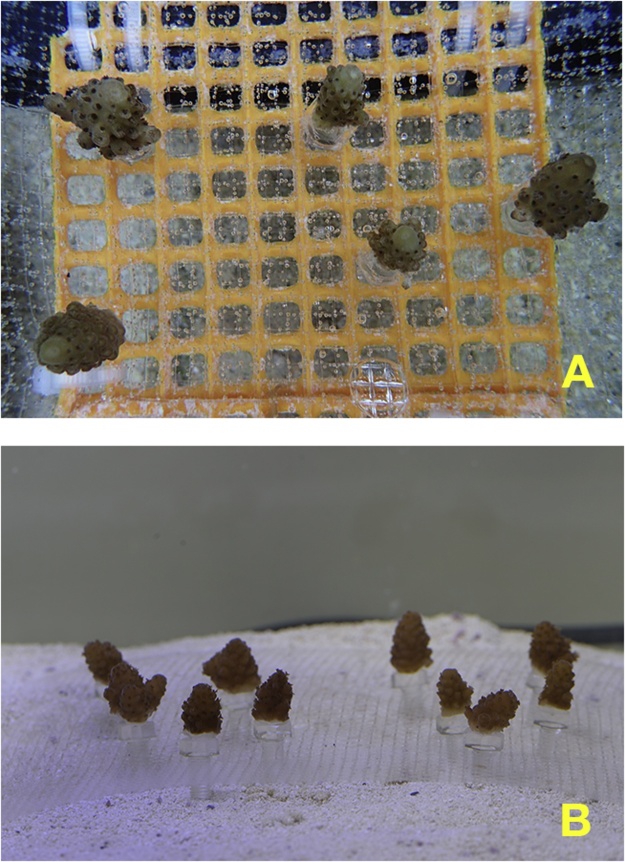
Small branches of *Acropora digitifera* transferred to the mesh substrate: in an aquarium with continuous supply of seawater at Sesoko Station, University of the Ryukyus (A); in a small aquarium at the laboratory of Graduate School of Agriculture, Ehime University (B). The mesh substrate consists of two screen layers, a thermoplastic fluoropolymer screen and an ethylene-vinyl acetate (EVA) screen; EVA screen was detached from the holding net at (B).

This new method was also tested for its sturdiness by subjecting the fragmented corals to long-distance aerial transport 3 days after their attachment to the substrate. The coral fragments affixed to the mesh substrate were transported from Sesoko Station, University of the Ryukyus, to the laboratory of Graduate School of Agriculture, Ehime University, at Matsuyama, Ehime, Japan. Matsuyama is located 930 km north-east from Sesoko Station in central Japan. During the transport, the substrate with corals was kept in a tripled plastic bag (0.1 mm thickness) filled with about 14 L of seawater. The sealed plastic bag was kept in a portable cooler (30 L) (Daiwa Light Trunk SU 3000RJ; Globeride Inc., Kurume, Tokyo, Japan). A small plastic bag with about 1–2 kg of crashed ice was set above the plastic bag holding the coral fragments in the cooler. Temperature inside the portable cooler was monitored with a small portable digital thermometer (O-209BL; Dretec Co. Ltd., Koshigaya, Saitama, Japan) and a temperature logger (Tidbit v2; Onset Computer Corp., Bourne, MA, USA) placed in the cooler.

The survival rate of the corals 3 days after the attachment procedure was 98.7% in total (*n* = 76) (Table 2). The transport from Sesoko to Matsuyama lasted about 7 h, including 2 h of aerial travel. During the transport, the temperature inside the cooler was kept at <28 °C, except at the beginning of its transport during the second trial. All 75 individuals in the three trials survived the transportation, while four individuals were detached from the bolts (Table 2).

**Table 2:** Survival of *Acropora* spp. fragments attached to polycarbonate bolts using Bondic®.

Species	*n*	Survival 3 d after attachment at the marine laboratory	Survival after the transportation	Survival 7 d after the transportation to the inland laboratory
*Acropora digitifera*	10	10	10	10
*Acropora digitifera*	34	34	34^a^	33
*Acropora tenius*	32	31	31	29

a Four individuals were detached from the bolt during the transportation.

At the destination location at Ehime University, salinity of the seawater in the plastic bag was measured and the coral fragments were acclimated over a period of 2–3 h by increasing the salinity by 1 psu per hour. After the acclimation, the corals with the top screen (thermoplastic fluoropolymer screen) of the mesh substrate were transferred into a small aquarium (72 L) that was placed inside an incubator (Fig. 4B). The rearing method conducted at Ehime University was identical to that described by Hirayama et al. [5], except for the size of the aquarium. Five days after the transportation, the detached fragments were reattached to the bolts using Bondic®. The survival rate of the corals 7 days after the transportation was 96.0% (Table 2). The total survival rate 10 days since the attachment, including the transportation, was 94.7% (Table 2).

The improvement of coral aquaculture becomes all the more crucial as an alternative method to wild harvest for ornamental trade and pharmaceutical usage and for restoration of coral reefs [11,15,16]. In coral aquaculture, development of new functional colonies from smaller fragments cut from adult colonies is the most popular method of asexual propagation of corals; fragments for aquaculture are also attached to various solid substrates such as rock, concrete, plastic and others [15]. “Aragocrete,” a mixture of Portland cement and aragonite sand, is the most common substrate used by coral hobbyist [16]. In coral restoration studies, the earliest developed and most common method used is the transplantation of coral fragments in 46% of the studies, followed by collection of “corals of opportunity” (corals fragmented through disturbance) [18]. At the restoration site, attaching the coral fragments to hard substrates generally resulted in higher survival rate compared with that obtained by merely placing the fragments onto the seafloor [18]. Epoxy was the most used for coral fragment attachments (in 28% of the studies), followed by cable ties (18%) and cements (8%) [18]. Thus, the present method is expected to be applied in coral aquaculture and restoration fields.

One of the photoinitiators in Bondic® is not reported in the Material Safety Data Sheet, whereas the acute toxicity of 2-hydroxy-2-methylpropiophenone, used for photoinitiators, to aquatic organisms is at 0.64 mg/L in EC_50_ 72 h for green algae and 160 mg/L in LC_50_ 96 h for the golden orfe *Leuciscus idus* [26]. The toxicity of this chemical to fish is weak—*n*-heptane at 375 mg/L and *n*-hexane at 113 mg/L in LC_50_ 96 h for Mozambique tilapia *Tilapia mossambica* [27]. Recently, Rogers et al. [28] assembled a micromanipulator from 3D-printed components by applying Bondic® and used the micromanipulator to record signals from the anterior lateral line nerve in free-swimming toadfish *Opsanus tau*. Thus, the usage of Bondic® could be applied for attachment of a wide range of aquatic organisms to substrate and/or experimental apparatus in various kinds of experiments.

In conclusion, the present study indicates that the use of Bondic® provides an inexpensive and instant method for attaching corals to experimental bases. In addition to ecotoxicity experiments, the present method would contribute to the development of aquaculture and aquarium exhibition of hermatypic corals and to the coral reef restoration.
